# A Case Report on the Utilization of a Hemostasis Analyzer System in the Management of a Patient With Essential Thrombocythemia

**DOI:** 10.7759/cureus.63787

**Published:** 2024-07-03

**Authors:** Tomohiro Nakajima, Kei Mukawa, Yutaka Iba, Yu Iwashiro, Nobuyoshi Kawaharada

**Affiliations:** 1 Cardiovascular Surgery, Sapporo Medical University, Sapporo, JPN

**Keywords:** aortic valve replacement, hemostasis, cardiopulmonary bypass, essential thrombocythemia, hemostasis analyzer

## Abstract

Essential thrombocythemia (ET) is a myeloproliferative neoplasm characterized by persistent elevation of platelet count due to abnormal proliferation of megakaryocytes. While some cases may be asymptomatic, the condition is associated with an increased risk of complications such as thrombosis and bleeding tendencies, necessitating appropriate management tailored to individual cases. Hemostasis analyzer systems are automated analytical devices designed for comprehensive evaluation of blood coagulation function. These systems enable rapid and accurate measurement of multiple parameters, including coagulation time, platelet function, and fibrin formation, thus facilitating a holistic assessment of hemostatic function. A 76-year-old male patient presented to our hospital. At the age of 65, he received treatment for promyelocytic leukemia and achieved remission. At 75 years, he developed leukocytosis, thrombocytosis, and progressive anemia. A comprehensive examination, including bone marrow biopsy and genetic testing, revealed a JAK2 mutation, leading to the diagnosis of ET. At the age of 76 years, he complained of chest discomfort during exertion. Further investigation revealed severe aortic valve stenosis and two-vessel coronary artery disease. The patient underwent aortic valve replacement and three-vessel coronary artery bypass grafting. A hemostasis analyzer system was used to monitor coagulation function throughout the procedure. Compared with the normal range, his coagulation profile showed a tendency toward hypercoagulability. Intraoperative and postoperative transfusions were performed as required. The patient's postoperative course was uneventful without any complications related to bleeding or thrombosis.

## Introduction

Essential thrombocythemia (ET) is a subtype of myeloproliferative neoplasm (MPN), a hematopoietic tumor characterized by excessive platelet production that leads to thrombotic and hemorrhagic complications [1]. This disease has a complex pathophysiology with contradictory tendencies, including a propensity for thrombosis and bleeding diathesis, both of which stem from persistent peripheral blood thrombocytosis and platelet dysfunction. The prognosis is largely determined by these secondary complications. Reports of artificial valve replacement and coronary artery bypass grafting in patients with ET are extremely rare, with concerns regarding intraoperative bleeding tendencies and postoperative thrombosis due to the nature of this disease [2]. Herein, we report a case of aortic valve and coronary artery stenosis complicated by ET, which was successfully treated with bioprosthetic aortic valve replacement and coronary artery bypass grafting [3].

## Case presentation

A 76-year-old man with a history of promyelocytic leukemia, who was treated and achieved remission at the age of 65 years, was under outpatient follow-up. At the age of 75 years, blood tests revealed leukocytosis, thrombocytosis, and anemia. Considering the possibility of leukemia relapse or myeloproliferative disorder, comprehensive evaluations including bone marrow biopsy and genetic testing were performed. Bone marrow findings were normal, but genetic testing revealed a JAK2 mutation, leading to the diagnosis of ET. Given the high thrombotic risk associated with this subtype, the patient was administered 100 mg aspirin daily.

**Figure 1:**
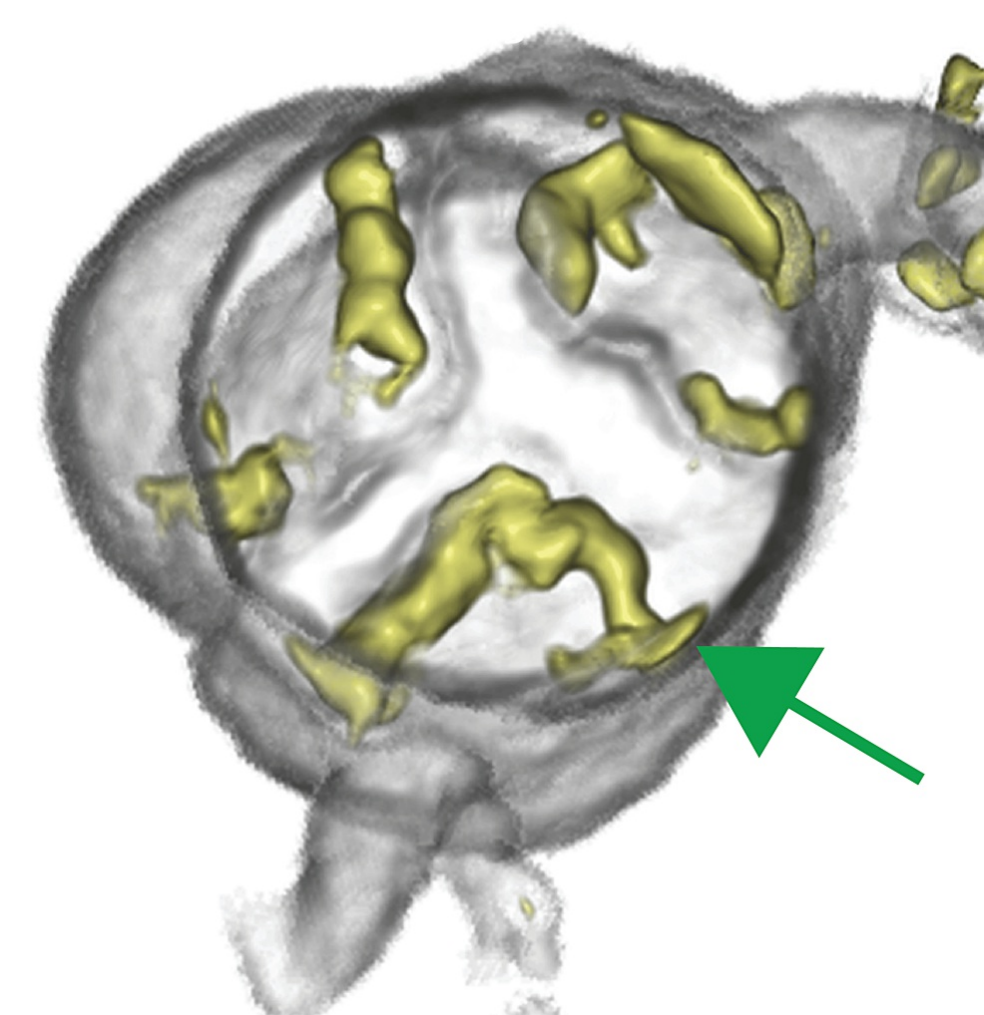
Preoperative images of 3D enhanced computed tomography The aortic valve was observed from the cephalic side. This figure demonstrates the preoperative state of the aortic valve with tricuspid morphology and severity (green arrow).

At the age of 76 years, he developed exertional dyspnea. Blood tests showed leukocytosis (16,500/μL), mild anemia (hemoglobin 9.3 g/dL), and thrombocytosis (933 × 10³/μL). Echocardiography revealed severe aortic valve stenosis (0.78 cm²; mean PG, 40.8 mmHg; and aortic valve orifice area 0.83 cm²) (Figure 1).

**Figure 2:**
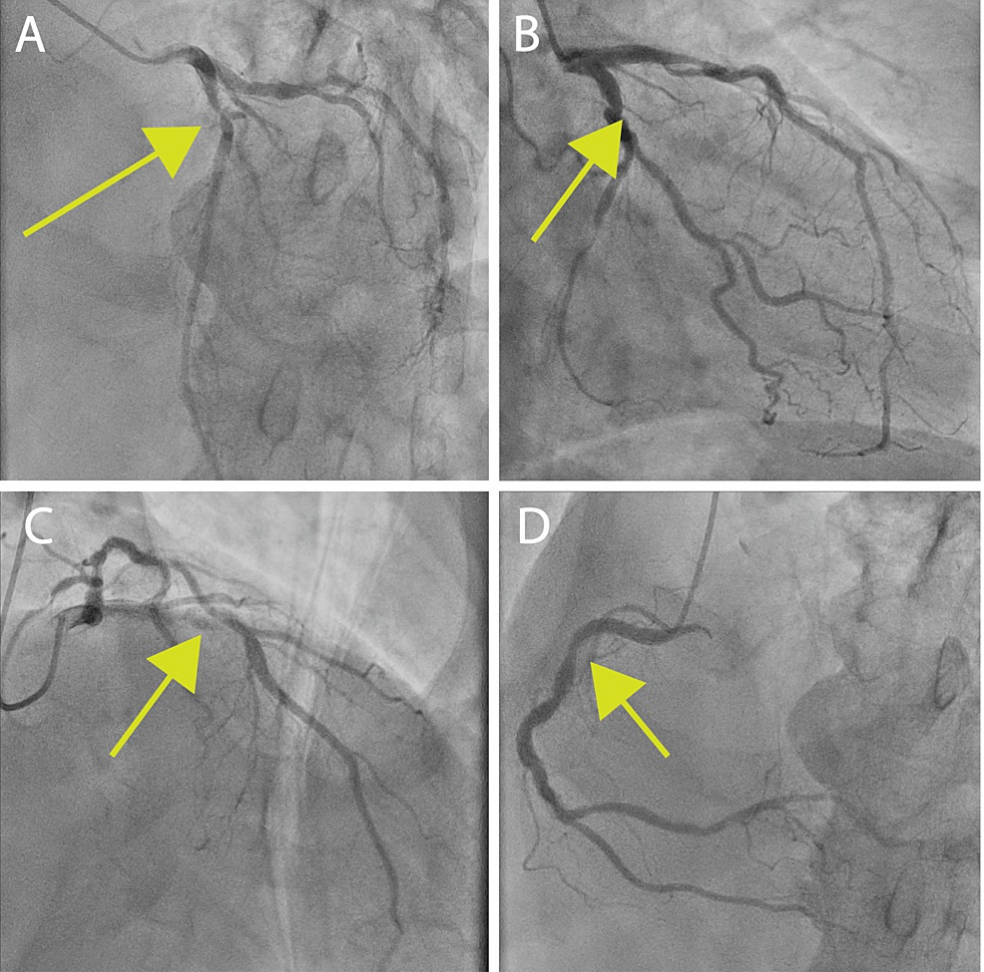
Preoperative coronary angiography (A) Left anterior descending artery (LAD) with 90% stenosis (green arrow). (B) Left circumflex artery (LCx) with 90% stenosis (green arrow). (C) Diagonal branch with 50% stenosis (green arrow). (D) Right coronary artery branch with 25% stenosis (green arrow).

Coronary angiography revealed 90% stenosis of the left anterior descending artery (LAD), 90% stenosis of the left circumflex artery (LCx), and 50% stenosis of the diagonal branches (Figures 2A-2C). Surgical intervention was deemed necessary, and bioprosthetic aortic valve replacement with coronary artery bypass grafting (left internal thoracic artery (LITA)-LAD; SVG-diagonal branch-posterolateral branch) was planned. Following the hospital safety protocol, aspirin was discontinued one week preoperatively and heparin bridging was initiated. In consultation with anesthesiologists, thromboelastography 6s (TEG6s, Haemonetics, Braintree, MA, USA) was employed intraoperatively to monitor coagulation status.

**Figure 3:**
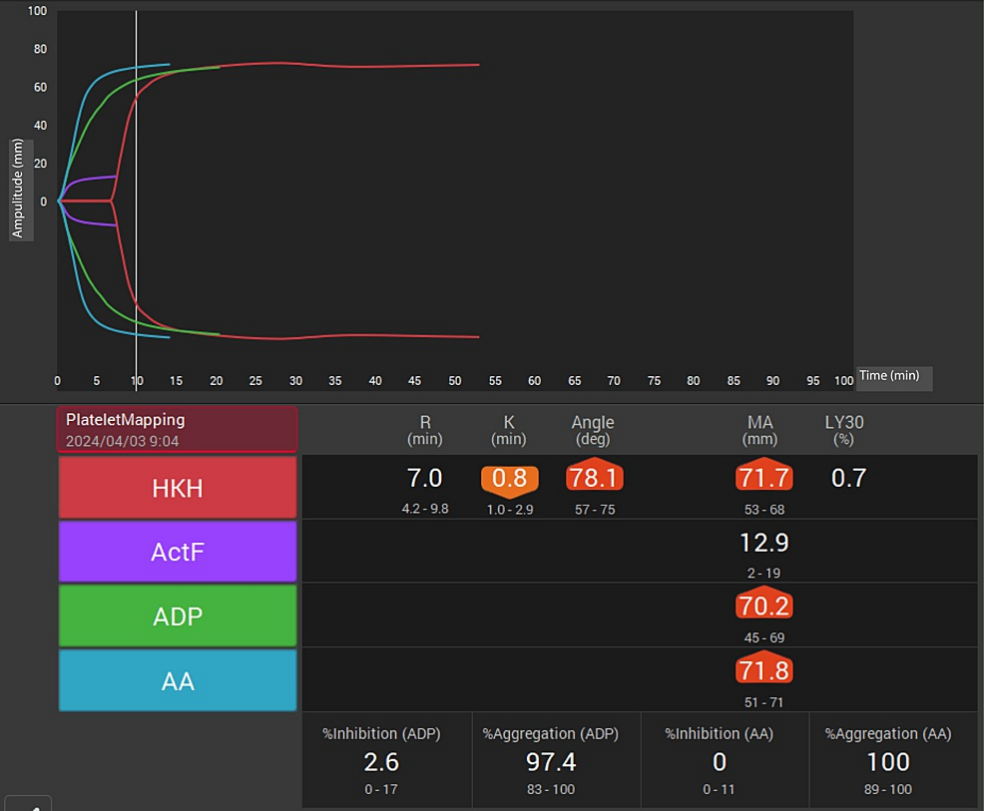
Preoperative thromboelastography (TEG6s) with HKH assay Preoperative coagulation profile using TEG6s with HKH assay This assay provides a comprehensive assessment of clot formation, strength, and stability. The overall clot strength (HKH-MA) is 71.7 mm (reference range: 52–70 mm) and fibrinogen contribution to clot strength (ActF-MA) is 12.9 mm (reference range: 7–22 mm).

Preoperative TEG6s measurements (MAHKH and early parameters: R, K, and angle) were obtained under general anesthesia (Figure 3). Both HKH-MA and ACT-F were above the reference range, indicating adequate overall clot strength. Following median sternotomy, LITA and left great saphenous vein were harvested. Cardiopulmonary bypass (CPB) was performed with an ascending aortic inflow and bicaval venous drainage. After aortic cross-clamping and antegrade cardioplegia, cardiac arrest was maintained with retrograde cardioplegia every 25 min. The posterior lateral branches of the left circumflex coronary artery and diagonal branch were anastomosed with a saphenous vein graft, followed by LITA-LAD anastomosis. The tricuspid aortic valve was excised, the annular calcification was debrided, and a 23 mm Inspiris bioprosthesis was implanted. After aortic closure and proximal SVG anastomosis, the aortic cross-clamp was released.

**Figure 4:**
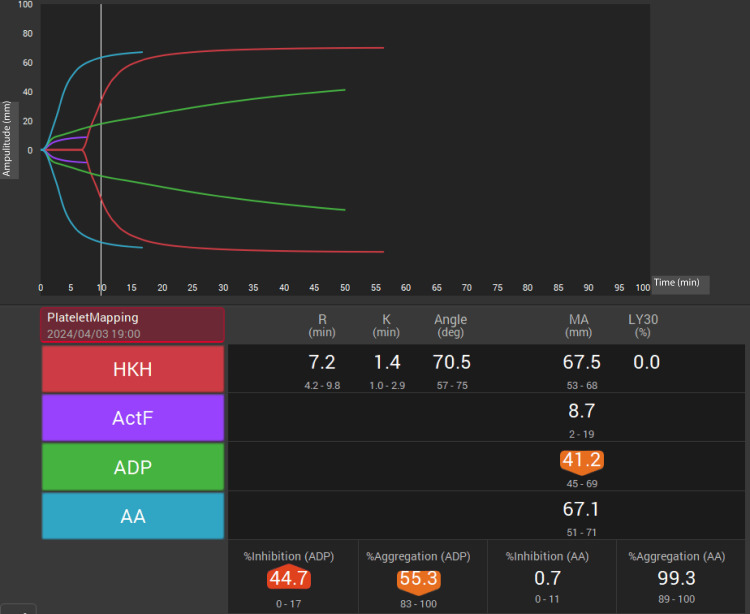
Postcardiopulmonary bypass thromboelastography (TEG6s) with HKH assay Postcardiopulmonary bypass (CPB) coagulation profile using TEG6s in the HKH assay. The assay was performed prior to intensive care unit transfer. Overall, clot strength (HKH-MA) is 67.5 mm (reference range: 52–70 mm)Fibrinogen contribution to clot strength (ActF-MA) is 8.7 mm (reference range: 7–22 mm). Platelet function (ADP-MA) is 41.2 mm (reference range: 45–69 mm).

During CPB weaning, repeated TEG6s measurements showed high HKH-MA and ACT-F, but low ADP-MA (42.2 mm; reference range: 45-69 mm), suggesting platelet dysfunction. Post-CPB, 10 units of FFP and 20 units of platelets were transfused. Hemostasis was achieved without difficulty. Preclosure TEG6s again showed low ADP-MA (41.2 mm), prompting an additional 20 units of platelets (Figure 4). Aortic cross-clamp, CPB, and total operative times were 224, 307, and 524 min, respectively.

Postoperatively, bleeding in the intensive care unit was minimal. The total perioperative transfusion included 1,120 mL of RBCs, 960 mL of FFP, and 400 mL of platelets. On postoperative day 1, heparin infusion (10,000 units/day), aspirin, and warfarin (target PT-INR 2.0) were initiated. Postoperative echocardiography revealed an effective aortic valve area of 1.75 cm², and graft patency was confirmed angiographically. Predischarge blood tests were similar to preoperative values (WBC 16,400/μL, Hb 8.5 g/dL, and platelets 682 × 10³/μL). On day 21, the patient experienced lower gastrointestinal bleeding with progressive anemia progression (PT-INR 2.01 and stable platelets), attributed to an ascending colon diverticulum, and was successfully treated with endoscopic clipping. The patient was transferred to another hospital 28 days postoperatively.

## Discussion

ET is a rare disorder within the spectrum of chronic MPNs, characterized by persistent peripheral blood thrombocytosis and platelet dysfunction, leading to a paradoxical tendency for both thrombosis and hemorrhage [4,5]. Here, we report a case of aortic valve and coronary artery stenosis complicated by ET managed with bioprosthetic aortic valve replacement and coronary artery bypass grafting.

Reports on open heart surgery in patients with ET are scarce. Gurrieri et al. documented a 28% perioperative complication rate in 25 ET patients undergoing cardiac surgery, with 80% of those experiencing complications having preoperative platelet counts exceeding 800 × 10³/μL [2]. The preoperative platelet count of our patient was 933 × 10³/μL, suggesting a heightened predisposition to complications.

In this case, we used the MAHKH and early parameters of the HKH assay to guide transfusion decisions perioperatively [6]. Preoperative assessments indicated adequate coagulation function, consistent with the patient's JAK2 mutation-positive status, a subtype associated with a high thrombotic risk. Postoperatively, the HKH assay revealed platelet dysfunction, prompting additional platelet transfusions, after which no pericardial bleeding occurred.

Three weeks after surgery, the patient developed anemia due to lower gastrointestinal bleeding [7]. With a therapeutic range PT-INR and stable platelet count, this event was more likely attributable to an ascending colon diverticulum than to ET or postoperative sequelae.

## Conclusions

Herein, we present the case of a patient with ET who underwent aortic valve replacement and coronary artery bypass grafting. Intraoperative coagulation function was assessed using TEG6s, which enable targeted transfusion therapy. Lower gastrointestinal bleeding on postoperative day 21 was not associated with ET.
